# Brain Volume of the Newly-Discovered Species *Rhynchocyon udzungwensis* (Mammalia: Afrotheria: Macroscelidea): Implications for Encephalization in Sengis

**DOI:** 10.1371/journal.pone.0058667

**Published:** 2013-03-13

**Authors:** Jason A. Kaufman, Gregory H. Turner, Patricia A. Holroyd, Francesco Rovero, Ari Grossman

**Affiliations:** 1 Department of Anatomy, Midwestern University, Glendale, Arizona, United States of America; 2 Center for Preclinical Imaging, Barrow Neurological Institute, Phoenix, Arizona, United States of America; 3 Museum of Paleontology, University of California, Berkeley, California, United States of America; 4 Sezione di Biodiversità Tropicale, Museo delle Scienze, Trento, Italy; 5 School of Human Evolution and Social Change, Arizona State University, Tempe, Arizona, United States of America; University of Lethbridge, Canada

## Abstract

The Gray-faced Sengi (*Rhynchocyon udzungwensis*) is a newly-discovered species of sengi (elephant-shrew) and is the largest known extant representative of the order Macroscelidea. The discovery of *R. udzungwensis* provides an opportunity to investigate the scaling relationship between brain size and body size within Macroscelidea, and to compare this allometry among insectivorous species of Afrotheria and other eutherian insectivores. We performed a spin-echo magnetic resonance imaging (MRI) scan on a preserved adult specimen of *R. udzungwensis* using a 7-Tesla high-field MR imaging system. The brain was manually segmented and its volume was compiled into a dataset containing previously-published allometric data on 56 other species of insectivore-grade mammals including representatives of Afrotheria, Soricomorpha and Erinaceomorpha. Results of log-linear regression indicate that *R. udzungwensis* exhibits a brain size that is consistent with the allometric trend described by other members of its order. Inter-specific comparisons indicate that macroscelideans as a group have relatively large brains when compared with similarly-sized terrestrial mammals that also share a similar diet. This high degree of encephalization within sengis remains robust whether sengis are compared with closely-related insectivorous afrotheres, or with more-distantly-related insectivorous laurasiatheres.

## Introduction

The Macroscelidea – or sengis – are small-bodied insectivorous mammals notable for their well-developed proboscis and robust hindlimb musculature. Their unique combination of physical, behavioral, and life history traits have been described as a ‘micro-cursorial adaptive syndrome’ [1] which includes small body size (<1 kg), a unique, highly cursorial locomotion, primarily myrmecophagous insectivory, relatively exposed sheltering habits, social monogamy, precocial litters, and female absentee neonatal care [2], [3]. It has been proposed that this suite of traits enables sengis to occupy extremes of terrestrial habitats ranging from arid deserts to closed-canopy forests [1].

Morphological studies have traditionally included sengis in the polyphyletic group ‘Insectivora’ (with shrews, hedgehogs, moles, golden moles, tenrecs, and solenodons). Such morphological studies highlight the adaptive similarities among small-bodied insectivoran mammals irrespective of phylogeny. These similarities include small body size, shared features of the dentition, and relatively small brain size [4]. However, more recent studies [5], [6] distinguished them from other insectivores, and recent molecular studies established the Macroscelidea as part of the supercohort Afrotheria, a monophyletic group with a very long evolutionary history [7], that contains five other orders: Proboscidea (elephants), Sirenia (manatees and dugongs), Hyracoidea (hyraxes), Tubulidentata (aardvarks), and Tenrecoidea (tenrecs and golden moles) [8]-[11]. Sengis are therefore relatively well- understood in terms of taxonomic position, behavioral ecology, and general morphology, but very little is known about their neuroanatomy.

**Figure 1:**
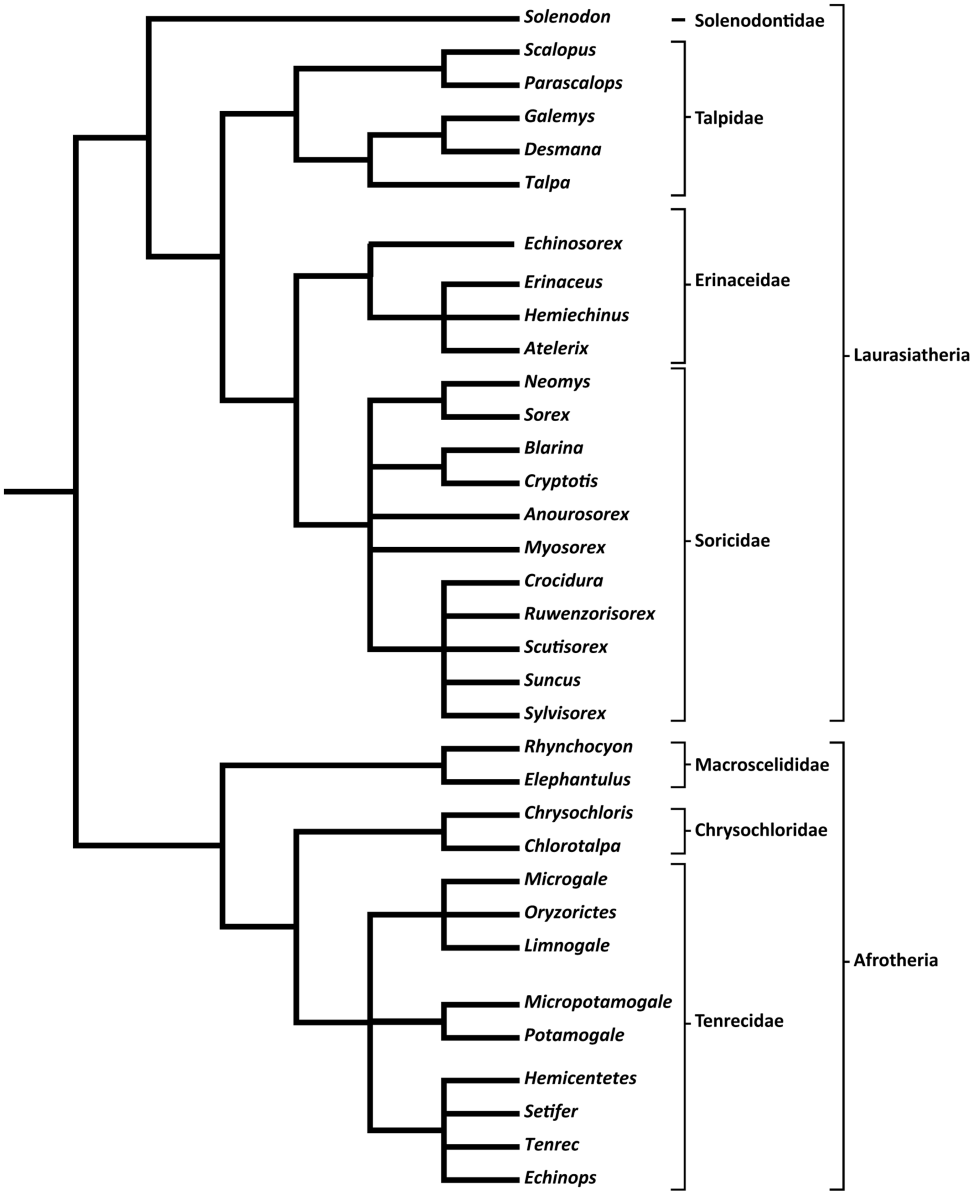
Phylogeny of genera included in the present analysis. A dendrogram illustrating the phylogenetic relationships among the genera investigated in the present study [11], [13]-[18].

Recently, a new species of giant sengi (*Rhynchocyon udzungwensis*) was discovered in Tanzania [12]. Prior to dissection of one of the specimens, we were able to perform magnetic resonance imaging (MRI) scans in order to measure the brain volume of the new species. In light of the taxonomic repositioning of sengis and the limited number of published data on sengi brain size, the discovery of *Rhynchocyon udzungwensis* provides an opportunity to re-analyze the relationship between brain size and body size in sengis. Thus, the goal of this study is to analyze brain/body allometry within sengis and compare this pattern of brain scaling to that of similarly-sized insectivore-grade terrestrial mammals. Here we compare sengis with closely-related afrotherian insectivorous tenrecs (Tenrecidae) and golden moles (Chrysochloridae), as well as more-distantly related laurasiatherian insectivores including Solenodontidae, Talpidae, Erinaceidae, and Soricidae. We are interested in determining 1) whether *R. udzungwensis* is similar to other sengis in its relative brain size, and 2) whether the relative brain sizes of sengis overlap that of other insectivore-grade terrestrial mammals. Figure 1 illustrates the phylogenetic relationships of the taxa used in this study [11], [13]-[18].

## Materials and Methods

Brain mass was measured from a preserved adult specimen (MTSN 8069) of *R. udzungwensis* described by Rovero et al. [12] as a partially-eaten carcass abandoned by a raptor. The carcass was collected under permit from the Tanzania Commission for Science and Technology [12]. Although parts of the pelvis and hindlimb had been consumed, the head was intact and well-preserved. To estimate brain volume we performed high-resolution 3D spin echo scans (TR = 81.6 ms, TE = 21.7 ms, Matrix = 512×512×256, Voxel size = 270 µm×270 µm×390 µm) using a 7-Tesla high-field MR imaging system (Bruker Biosystems). After scanning, the brain was manually segmented using Amira (Visage Imaging) and its volume was computed by multiplying the number of voxels contained in the segmented volume by the voxel size. Brain mass was then computed by multiplying the brain volume by the specific gravity of brain tissue (1.036 g/cm^3^).

The condition of the carcass did not allow for sex determination or direct measurement of body mass. In their description of the species – including physical measurements as well as visual sightings – Rovero et al. note a lack of size dimorphism in *R. udzungwensis* and a lack of sexual dimorphism in general with the exception of canine length [12]. For this study, we therefore use the average body mass of four captured adults (one female and three males) as reported by Rovero et al. (mean = 710 g, standard deviation = 20 g) [12].

The new *R. udzungwensis* data point was then compiled into a dataset containing previously-published brain- and body-size data for four other species of Macroscelididae as well as 54 other species of insectivore-grade terrestrial mammals, including the afrotherian insectivorous Tenrecidae and Chrysochloridae, as well as the laurasiatherian insectivorous Solenodontidae, Erinaceidae, Soricidae, and Talpidae [19]-[22]. The dataset used for our analysis is presented in Table 1.

**Table 1:** Brain and body size data.

Family	Genus	Species	Body mass (g)	Brain mass (mg)	Source
Solenodontidae	*Solenodon*	*paradoxus*	672	4723	[21]
Tenrecidae	*Tenrec*	*ecaudatus*	852	2588	[21]
Tenrecidae	*Setifer*	*setosus*	237	1516	[21]
Tenrecidae	*Hemicentetes*	*semispin*	116	839	[21]
Tenrecidae	*Echinops*	*telfairi*	87.5	623	[21]
Tenrecidae	*Oryzorictes*	*talpoides*	44.2	580	[21]
Tenrecidae	*Microgale*	*cowani*	15.2	420	[22]
Tenrecidae	*Limnogale*	*mergulus*	92	1150	[21]
Tenrecidae	*Microgale*	*dobsoni*	31.9	557	[21]
Tenrecidae	*Microgale*	*talazaci*	48.2	766	[21]
Tenrecidae	*Micropotamog*	*lamottei*	64.2	800	[21]
Tenrecidae	*Micropotamog*	*ruwenzorii*	96.8	1134	[21]
Tenrecidae	*Potamogale*	*velox*	618	4152	[21]
Chrysochloridae	*Chlorotalpa*	*stuhlmanni*	40.2	736	[21]
Chrysochloridae	*Chrysochlori*	*asiatica*	49	700	[21]
Erinaceidae	*Atelerix*	*algirus*	736	3264	[21]
Erinaceidae	*Erinaceus*	*europaeus*	849	3367	[21]
Erinaceidae	*Hemiechinus*	*auritus*	235	1880	[21]
Erinaceidae	*Echinosorex*	*gymnurus*	823	6084	[21]
Soricidae	*Sorex*	*alpinus*	11	262	[21]
Soricidae	*Sorex*	*cinereus*	5.2	168	[21]
Soricidae	*Sorex*	*fumeus*	8.4	241	[21]
Soricidae	*Sorex*	*minutus*	4.4	115	[21]
Soricidae	*Sorex*	*araneus*	10.2	216	[21]
Soricidae	*Microsorex*	*hoyi*	2.6	96	[21]
Soricidae	*Neomys*	*anomalus*	11.6	282	[21]
Soricidae	*Neomys*	*fodiens*	15.3	328	[21]
Soricidae	*Blarina*	*brevicauda*	19.7	393	[21]
Soricidae	*Cryptotis*	*parva*	9.9	245	[21]
Soricidae	*Anourosorex*	*squamipes*	20.1	389	[21]
Soricidae	*Crocidura*	*attenuata*	9.3	209	[21]
Soricidae	*Crocidura*	*flavescens*	29.3	414	[21]
Soricidae	*Crocidura*	*giffardi*	82	545	[21]
Soricidae	*Crocidura*	*hildegardeae*	10.2	213	[21]
Soricidae	*Crocidura*	*occidentalis*	28	440	[22]
Soricidae	*Crocidura*	*russula*	11.1	197	[21]
Soricidae	*Crocidura*	*suaveolens*	10.3	190	[21]
Soricidae	*Crocidura*	*jacksoni*	12.7	250	[21]
Soricidae	*Crocidura*	*leucodon*	13.5	190	[21]
Soricidae	*Suncus*	*etrescus*	1.9	62	[21]
Soricidae	*Suncus*	*murinus*	33.8	383	[21]
Soricidae	*Scutisorex*	*somereni*	63.4	640	[21]
Soricidae	*Sylvisorex*	*granti*	3.9	165	[21]
Soricidae	*Sylvisorex*	*megalura*	5.5	188	[21]
Soricidae	*Ruwenzorisor*	*suncoides*	18.2	370	[21]
Soricidae	*Myosorex*	*babaulti*	17	360	[21]
Talpidae	*Talpa*	*europaea*	82.1	1024	[21]
Talpidae	*Talpa*	*micrura*	41.4	816	[21]
Talpidae	*Parascalops*	*breweri*	53.8	880	[21]
Talpidae	*Scalopus*	*aquaticus*	115	1310	[21]
Talpidae	*Desmana*	*moschata*	443	4000	[21]
Talpidae	*Galemys*	*pyrenaicus*	59.7	1329	[21]
Macroscelididae	*Elephantulus*	*fuscipes*	57	1330	[22]
Macroscelididae	*Elephantulus*	*myurus*	45.1	1270	[19]
Macroscelididae	*Rhynchocyon*	*stuhlmanni*	490	6100	[22]
Macroscelididae	*Rhynchocyon*	*petersi*	471	5400	[20]
Macroscelididae	*Rhynchocyon*	*udzugwensis*	710	7131	Present Study

Dataset of body size and brain size for insectivorous mammals used in the present analysis.

To determine whether Macroscelidea as a group exhibit larger or smaller brains for a given body size than other insectivores, we performed reduced major axis (RMA) regression of log body mass on log brain mass and tested for differences in the RMA line fitted to Macroscelidea versus the RMA line fitted to other insectivores. Statistical tests were performed using the SMATR (Standardised Major Axis Tests & Routines) software toolkit [23]. In this method, differences in fitted regression lines are tested using the WALD test on residual scores (to detect differences in line elevation/intercept) and fitted scores (to detect shifts along a common slope) [23].

In order to control for statistical non-independence due to phylogeny, we performed additional tests using a phylogenetic generalized least squares (PGLS) regression model with Pagel’s lambda [24], [25]. PGLS analyses were conducted with the caper software package [26] in the R computing environment [27] using branch lengths from Bininda-Emonds et al. [28].

## Results

**Figure 2:**
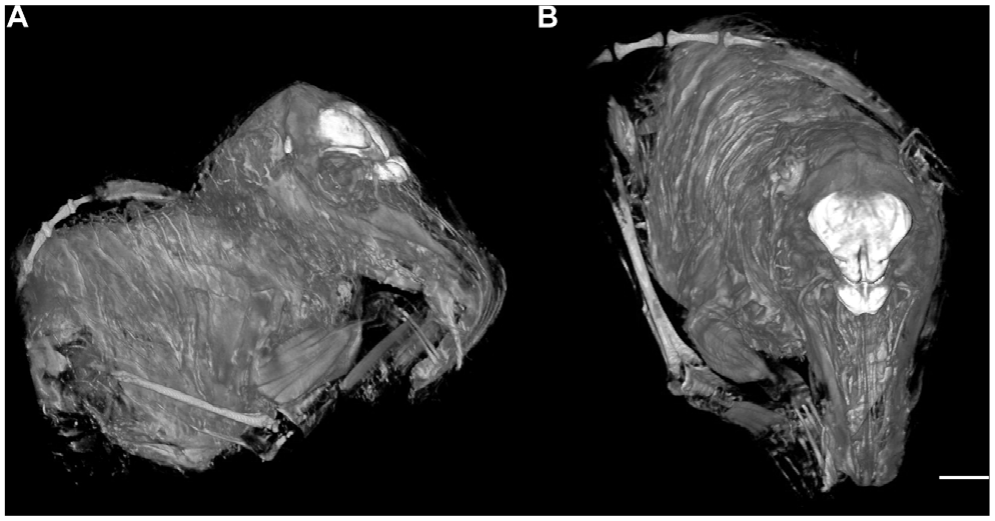
Maximum intensity projections of the *R. udzungwensis* MRI. Two views of the *R. udzungwensis* MRI visualized as maximum intensity projections with the brain highlighted in white. A) Antero-lateral oblique view. B) Superior view, scale bar = 5 cm.

**Figure 3:**
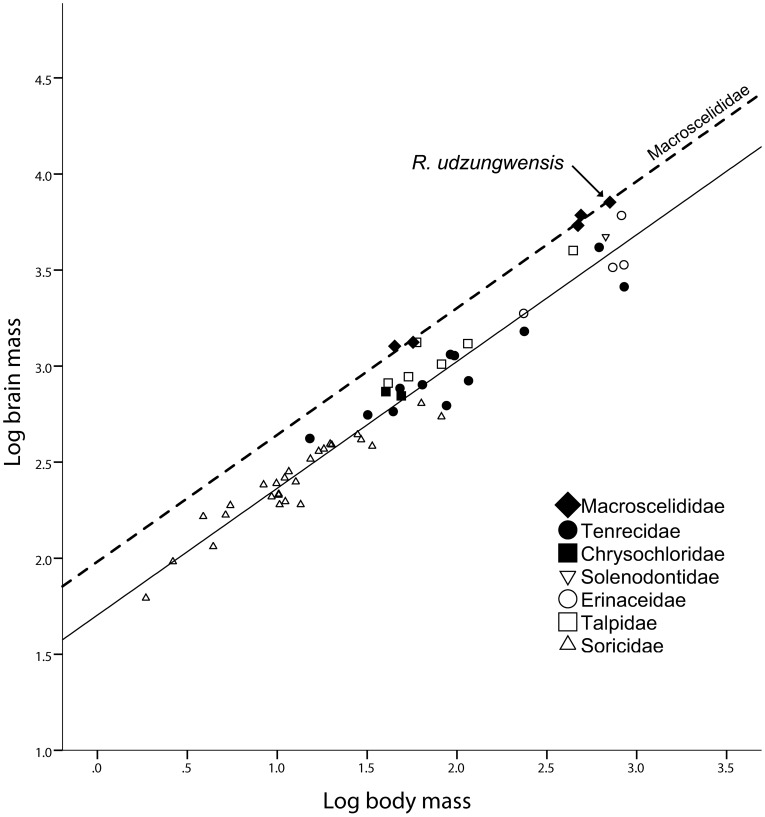
Brain-body allometry in Macroscelididae vs. other insectivores. A scatterplot of log body mass on log brain mass in which the RMA line (dashed) for Macroscelididae (n = 5) is compared to the RMA line (solid) describing other insectivores (n = 52). The slopes of the two lines are statistically indistinguishable (common slope  = 0.66; 95% CI: 0.63–0.70). Residual axis scores indicate that the best-fit line describing Macroscelididae has a significantly larger y-intercept than the line describing other insectivores (X^2^ = 142.36, *p*<0.001).

Segmentation of the *R. udzungwensis* MRI (Figure 2) yielded a brain volume of 6.883 cc^2^. When multiplied by the specific gravity of brain tissue, the brain mass is calculated to be 7.131 g. This represents the largest brain in the present dataset, followed by the brains of *Echinosorex* and two other *Rhynchocyon* species. With a body mass of 710 g, *R. udzungwensis* is surpassed in body size by several other species of Erinaceidae and Tenrecidae. When the brain size of *R. udzungwensis* is compared with the four other species of Macroscelidea in the dataset, the *R. udzungwensis* datapoint falls on the allometric trend line defined by the two smaller-bodied *Elephantulus* species and the two larger-bodied *Rhynchocyon* species (Figure 3). Although the sample size is small, this indicates that the brain mass of *R. udzungwensis* is consistent with what would be expected in a sengi of its body mass.

RMA regression of body mass on brain mass for all species yields a best-fit line with a slope of 0.71 (n = 57, *R^2^* = 0.94, *p*<0.001; 95% CI: 0.67–0.76). For inter-specific tests, we compare brain allometry in: 1) sengis versus all other insectivores, 2) sengis versus other afrotherian insectivores only, 3) sengis versus laurasiatherian insectivores, and 4) afrotherian insectivores (including sengis) versus laurasiatherian insectivores. The results of each of these comparisons are summarized in Table 2.

**Table 2:** Summary of results.

Comparison	Test for heterogeneity of slopes	Common slope (95% CI)	WALD test for difference in elevation/intercept	WALD test for shift along common slope
sengis vs. other insectivores	*p* = 0.336	0.66 (0.63–0.70)	X^2^ = 142.36, *p*<0.001	X^2^ = 9.847, *p* = 0.002
sengis vs. other afrotherian insectivores only	*p* = 0.163	0.65 (0.55–0.70)	X^2^ = 76.94, *p*<0.001	X^2^ = 3.632, *p* = 0.057
sengis vs. laurasiatherian insectivores only	*p* = 0.229	0.67 (0.64–0.72)	X^2^ = 110.471, *p*<0.001	X^2^ = 11.846, *p* = 0.001
afrotherian insectivores vs. laurasiatherian insectivores	*p* = 0.626	0.70 (0.65–0.74)	X^2^ = 0.864, *p* = 0.353	X^2^ = 13.829, *p*<0.001

Statistical comparisons of brain/body allometry among the insectivores in the present sample. Results indicate that sengis have relatively larger brains for a given body size compared with other insectivore-grade mammals.

When the sample is grouped according to Macroscelidea (n = 5) versus all other insectivores (n = 52), the test for heterogeneity of slopes indicates that the slopes of the two lines are statistically indistinguishable. The common slope for the two lines is computed to be 0.66 (95% CI: 0.63–0.70). The WALD test for comparisons of lines with common slopes indicates a significant difference in elevation (y-intercept) between the two RMA lines. Comparisons of residual axis scores indicate that the best-fit RMA line for Macroscelidea has a significantly higher elevation than the best-fit line for other insectivores (Figure 3, Table 2). Comparisons of the fitted axis scores also indicate a positive shift along the common slope for the Macroscelidea, reflecting the relatively large body size of Macroscelidea within the insectivore range.

We repeated this comparison using the PGLS model to control for phylogeny. Pagel’s lambda was 0.880, indicating a strong phylogenetic signal in the model. The difference in y-intercept between sengis and other insectivores remained statistically significant (*t* = 2.645, *p* = 0.011) while the slopes remained statistically indistinguishable. This result indicates that Macroscelidea have larger brains for a given body mass compared with other insectivores even when controlling for phylogenetic history.

Comparisons of subsets of the data further elucidate this pattern (Table 2). When afrotherian insectivores (including sengis) are compared with laurasiatherian insectivores, the two slopes are statistically indistinguishable and there is no statistical difference between the elevation of the allometric lines defining the two groups. Fitted axis scores indicate a positive shift along the common slope for Afrotheria, reflecting their comparatively large body size distribution. However, when sengis are compared with other afrotherian insectivores or with laurasiatherian insectivores, the best-fit RMA line for Macroscelidea has a significantly higher elevation than the best-fit line for either of these subgroups.

Taken together, these results indicate a robust separation between the allometric clustering of Macroscelidea versus other insectivores. Although the afrotherian insectivores as a group (including sengis) do not differ statistically in brain allometry compared to laurasiatherian insectivores, the Macroscelidea are shown to have larger brains for a given body mass compared with other insectivores in the dataset. This separation remains significant when sengis are compared with laurasiatherian insectivores, as well as with other afrotherian insectivores.

## Discussion

The recent discovery of new extant species of sengi has increased the number of known species of Macroscelidea and more may yet be described [29]-[31]. Dumbacher and colleagues [31] recently elevated the two subspecies of *Macroscelides* to species level using a combination of genetic and morphological markers. In 2008, Smit et al. discovered *Elephantulus pilicaudus* during an investigation of genetic biogeography in South African sengis [30]. Also in 2008, Rovero et al. reported the discovery of *Rhynchocyon udzungwensis* from isolated high-elevation forests of Tanzania [12]. *R. udzungwensis* has the largest body mass of any extant sengi yet discovered. There is a paucity of data on brain size in macroscelideans, but in the present study we are able to compare the brain size of the newly-discovered *Rhynchocyon* specimen with data from four other species of Macroscelidea. Our analyses indicate that *R. udzungwensis* exhibits a brain mass that is within the confidence intervals of the regression line described by the small-bodied *Elephantulus* and the large-bodied *Rhynchocyon*. These results suggest a consistent pattern of brain allometry within Macroscelidea, although additional data collection on other sengis will be necessary in order to quantify this relationship more precisely.

Very little is known about brain allometry in Macroscelidea compared with other insectivores, especially following the taxonomic repositioning of sengis from ‘Insectivora’ into Afrotheria. Stephan et al. [21], [22] provide the most comprehensive dataset of brain- and body-size among insectivores (including size of individual brain structures). They report brain-size values for three species of Macroscelidea (incorporated here), but the authors recognized that Macroscelidea had likely been incorrectly placed within ‘Insectivora’ and therefore excluded the sengis from their analyses.

Our inter-specific comparisons using the new phylogenetic rubric indicate that macroscelideans have relatively large brains when compared with similarly-sized terrestrial mammals that also share a similar diet. This high degree of encephalization within sengis appears to hold whether sengis are compared with closely-related insectivorous afrotheres, or with more-distantly-related insectivorous laurasiatheres. In fact, the brain-body allometry of Macroscelidea may be more similar to larger-brained non-insectivorous groups such as Rodentia or Lagomorpha, rather than smaller-brained insectivores.

An alternative interpretation that sengis have relatively smaller bodies must also be considered. The earliest sengis are primarily known from dental specimens. Grossman and Holroyd [32] use published equations [33], [34] for reconstructing small mammal body mass from dental dimensions to reconstruct the body mass of early sengis. These reconstructions indicate that the earliest Macroscelideans such as *Chambius* [35], [36] and *Nementchatherium* [37] were similar in body size to modern macroscelidine sengis such as *Elephantulus* or *Petrodromus*. Early members of the modern sengi subfamilies Rhynchocyoninae (*Myorhynchocyon* [38], [39]) and Macroscelidinae (*Miosengi* [40]) are similar in size to their living relatives. Thus, there is little evidence to suggest that sengis underwent body size reduction during their evolution.

The functional significance of sengi encephalization remains unclear. But there are some suggestions from the literature that merit further study. Using electrophysiology, Dengler-Crish et al. found a large somatosensory representation of the proboscis, vibrissae, and tongue in the cortex of the South African sengi *Elephantulus edwardii* [41]. And using immunohistochemistry, Pieters et al. found cholinergic neurons present in the cochlear nucleus and both colliculi of the Eastern Rock sengi *Elephantulus myurus* that are not present in hyraxes, rodents, and primates, possibly suggesting an auditory adaptation for predator avoidance [19]. Additionally, Sherwood et al. found that that the giant elephant shrew *Rhynchocyon petersi* exhibits a high density of calretinin interneurons, a trait which they find to be derived from the stem mammal condition [20].

In relation to our analysis, sengis differ radically from other insectivores in their locomotor behavior, especially as it pertains to predator avoidance mechanisms. Modern sengis move very quickly and with notable agility [2], [3] by a unique cursorial/saltatorial mode of locomotion [2], [42] using their relatively longer hindlimbs to generate a bounding motion. By contrast, most other insectivore-grade mammals move comparatively slowly. Furthermore, sengis create and maintain a complex trail system that they use for escaping predators [43]-[45]. Perhaps in the future, the underlying neural mechanisms for these behaviors, and many others, will help to explain the pattern of sengi brain allometry observed here.
